# Strategies for the Covalent Anchoring of a BMP-2-Mimetic Peptide to PEEK Surface for Bone Tissue Engineering

**DOI:** 10.3390/ma16103869

**Published:** 2023-05-21

**Authors:** Leonardo Cassari, Annj Zamuner, Grazia Maria Lucia Messina, Martina Marsotto, Hao-chen Chang, Trevor Coward, Chiara Battocchio, Giovanna Iucci, Giovanni Marletta, Lucy Di Silvio, Monica Dettin

**Affiliations:** 1 Department of Industrial Engineering, University of Padova, Via Marzolo 9, 35131 Padova, Italy; leonardo.cassari@phd.unipd.it; 2 Department of Civil, Environmental, and Architectural Engineering, University of Padova, Via Marzolo 9, 35131 Padova, Italy; 3 Laboratory for Molecular Surface and Nanotechnology (LAMSUN), Department of Chemical Sciences, University of Catania and CSGI, Viale A. Doria, 6, 95125 Catania, Italy; 4 Department of Science, Roma Tre University, Via della Vasca Navale 79, 00146 Roma, Italy; 5 Faculty of Dentistry, Oral & Craniofacial Sciences, King’s College London, London SE1 9RT, UK

**Keywords:** PEEK, peptides, surface functionalization, BMP-2, human osteoblasts, 3D printing, bone tissue engineering

## Abstract

Researchers in the field of tissue engineering are always searching for new scaffolds for bone repair. Polyetheretherketone (PEEK) is a chemically inert polymer that is insoluble in conventional solvents. PEEK’s great potential in tissue engineering applications arises from its ability to not induce adverse reactions when in contact with biological tissues and its mechanical properties, which are similar to those of human bone. These exceptional features are limited by the bio-inertness of PEEK, which causes poor osteogenesis on the implant surface. Here, we demonstrated that the covalent grafting of the sequence (48–69) mapped on the BMP-2 growth factor (GBMP1α) significantly enhances the mineralization and gene expression of human osteoblasts. Different chemical methods were employed for covalently grafting the peptide onto 3D-printed PEEK disks: (a) the reaction between PEEK carbonyls and amino-oxy groups inserted in the peptides’ N-terminal sites (oxime chemistry) and (b) the photoactivation of azido groups present in the peptides’ N-terminal sites, which produces nitrene radicals able to react with PEEK surface. The peptide-induced PEEK surface modification was assessed using X-ray photoelectron measurements, while the superficial properties of the functionalized material were analyzed by means of atomic force microscopy and force spectroscopy. Live and dead assays and SEM measurements showed greater cell cover on functionalized samples than the control, without any cytotoxicity induction. Moreover, functionalization improved the rate of cell proliferation and the amount of calcium deposits, as demonstrated by the AlamarBlue™ and alizarin red results, respectively. The effects of GBMP1α on h-osteoblast gene expression were assayed using quantitative real-time polymerase chain reaction.

## 1. Introduction

The increased life expectancy in Western countries has given rise to higher demands for orthopedic implants in order to address bone and cartilage pathologies [1]. Traditionally, implants have been composed of metals because of their high resistance to oxidation, outstanding mechanical characteristics, and biocompatibility [2]. Nonetheless, the dissimilarity between the elastic modulus of these metals and that of cortical bone, combined with the discharge of metal ions from the implant surface, can cause issues and sometimes leads to implant malfunction. Furthermore, the majority of polymeric substances are unable to endure repeated loads without undergoing permanent deformation [3]. Polyetheretherketone (PEEK) has been found to be an exception to this. PEEK is a linear, semi-crystalline polymer that contains an aromatic backbone with 1,4-disubstituted phenyl groups separated by linkages of ether (-O-) and carbonyl (-CO-). PEEK’s biomechanical properties are comparable to human bones; these features highlighted PEEK as a potential polymeric substitute for conventional metal implants. Its elastic modulus (around 3.6 GPa) can be modified through additive inclusion, such as carbon reinforcement, making it comparable to the modulus of cortical bone (about 18 Gpa) [4,5,6]. In consideration of its exceptional mechanical properties, PEEK was proposed as a substitute for the metal components of orthopedic implants by the late 1990s. Its usage specifically extends to femoral prostheses and hip joints [7] and, in recent times, even dentists have valued PEEK, due to its biomechanical and aesthetic properties [8].

The scientific Interest in PEEK is increasing as a result of its potential use in 3D printing, since it is a thermoplastic polymer that can be shaped in its melting temperature range [9,10], around 343 °C, which is relatively high compared to other polymers in its class [4,10].

PEEK has shown high resistance to thermal degradation, organic and aqueous environment-related degradation, and biodegradation [11]. Consequently, PEEK implants are biocompatible, eliciting only mild inflammatory responses upon application [12]. Given their hydrophobic surface, PEEK implants are bio-inert, avoiding any protein adsorption and not promoting cell adhesion [13]. An enhancement of cellular interaction is desirable in orthopedic prostheses, especially for elderly patients or patients with multiple pathologies, where the osseointegration process is critical. This enables faster growth of new bone tissue, which can make direct contact with the implant’s surface. Consequently, the alteration of PEEK’s surface with the aim of promoting its recognition by cells through biochemical messages presents an attractive challenge that could further increase the positive features of this material [14], and this represents the aim of this work. 

In the literature, several physical methods for the surface modification of PEEK have been proposed: thermal spraying [15], pulsed laser treatment [16], ion sputtering [17], sandblasting [18], and electrochemical approaches [19]. All these methods may promote an increase in PEEK bioactivity, but they are challenging to perform and require complex equipment.

Previous studies have proposed two different methods for PEEK functionalization. In the work of Yakufu et al., cell adhesion or proliferation were promoted by anchoring signal sequences to the isocyanate or amino groups, previously introduced through the reduction of the carbonyl groups in PEEK into alcohol groups [20,21,22]. Alternatively, in our previous paper, the PEEK ketones were involved in a chemical reaction with amino-oxy groups introduced in adhesive sequences [23]. The covalent bond occurs in a simple procedure without the need for other reagents [24,25]. In this work, the functionalization via oxime was compared to another strategy that employs photoactivable bioactive peptides. This last method is more versatile since the photoactivated azido group can react with different groups of the PEEK’s surface and is more complex because it includes the photoactivation step [26]. Both the amino-oxy group and the azido group were introduced in the sequence of the fragment (48–69) of the BMP-2 protein (GBMP1α). GBMP1α is a bioactive peptide proven to be able to enhance osteogenic activities [27,28]. The results of the physico-chemical, mechanical, and biological characterization of the functionalized PEEK samples are shown and discussed below.

## 2. Materials and Methods

### 2.1. Materials

Merck Millipore (Burlington, MA, United States) provided acetic acid, acetone, acetonitrile, Fmoc-protected amino acids, Bis-Boc-aminooxy acetic acid (Aoa), dichloromethane (DCM), N,N-diisopropylethylamine (DIPEA), N,N-dimethylformamide (DMF), ethyl cyano(hydroxyimino)acetate (Oxyma Pure), methanol, Rink amide MBHA resin, triethylamine (TEA), triethylsilane (TES), and 2-(1H-benzotriazol-1-yl)-1,1,3,3-tetramethyluronium hexafluoro phosphate (HBTU). Biosolve (Valkenswaard, Holland) provided diethyl ether and trifluoroacetic acid (TFA). PanReac Appli-Chem (Darmstadt, Germany) provided 2-propanol for HPLC (aldehyde and ketone free), while Carlo Erba (Milan, Italy) provided ethanol. Iris Biotech GmbH (Marktredwitz, Germany) provided N-Methyl-2-pyrrolidone (NMP).

### 2.2. Synthesis of the Peptide Aoa-x-GBMP1α

The peptide Aoa-x-GBMP1*α* (sequence: H-Aoa-(7-aminoheptanoic acid)- Pro-Phe-Pro-Leu-Ala-Asp-His-Leu-Asn-Ser-Thr-Asn-His-Ala-Ile-Val-Gln-Thr-Leu-Val-Asn-Ser-NH_2_) was synthesized using solid-phase peptide synthesis (SPPS) on Rink amide MBHA resin with a Syro I synthesizer (Multi-syntech, Witten, Germany). The N-termini of all amino acids were protected with an Fmoc group and the side chains of His, Asn, and Gln were protected with Trt, Asp with OtBu, and Ser/Thr with tBu. A double coupling strategy was used for every amino acid addition. Five equivalents of each amino acid, five equivalents of activating agent HBTU/Oxima Pure, and ten equivalents of DIPEA, with respect to the resin reactive groups, were used for each coupling of 45 min. As a spacer (x) between the bioactive peptide sequence and the Aoa, 7-aminoheptanoic acid was introduced with the same procedure. Aoa was added to the sequence through a double coupling with five equivalents, in addition to five equivalents of activating agent HBTU/Oxima Pure and ten equivalents of collidine, with respect to the resin reactive groups, for each coupling of 45 min. After that, the peptide was cleaved from the resin and deprotected from the Bis-Boc of Aoa and the side chain-protecting groups using 4.75 mL TFA, 0.125 mL TES, and 0.125 H_2_O for 1.5 h at room temperature. The resin was filtered off. The solution was concentrated, and the crude peptide was precipitated with cold diethyl ether addition. The identity of the peptide was determined through mass spectrometry (theoretical mass = 2587.86 Da; experimental mass = 2587.81 Da; SCIEX TOF-TOF 4800 instrument, Foster City, CA, USA). The peptide was purified using RP-HPLC and characterized through analytical RP-HPLC using a Vydac C18 column (5 μm, 300 Å, 4.6 × 205 mm, Grace) in the following conditions: eluent A (0.05% TFA in MilliQ water); eluent B (0.05% TFA in 40% isopropanol and 60% MilliQ water); gradient, from 60 to 80% B in 40 min; flow rate, 0.5 mL/min; detection at 214 nm (retention time: 24.17 min).

### 2.3. Synthesis of the Peptide N_3_-x-GBMP1α

The peptide N_3_-x-GBMP1α (sequence: H-Phe(p-N_3_)-Gly-γAbu-Pro-Phe-Pro-Leu-Ala-Asp-His-Leu-Asn-Ser-Thr-Asn-Ser-Thr-Asn-His-Ala-Ile-Val-Gln-Thr-Leu-Val-Asn-Ser-NH_2_) was synthesized as reported for Aoa-x-GBMP1α synthesis. γAbu and Gly were introduced as a spacer (x) between the bioactive peptide sequence and Aoa (since the total length of the two molecules together was the same as 7-amino heptanoic acid). Finally, the sequence was enriched with an azido group on the N-terminus through a double coupling of Fmoc-p-azido-Phe-OH with the same protocol used for the other amino acids. Therefore, the deprotection of the peptide from the Fmoc occurred before the cleavage from the resin and deprotection from the side chain-protecting groups using 4.75 mL TFA, 0.125 mL TES, and 0.125 H_2_O for 90 min at room temperature. The crude peptide in solution was separated from the resin, concentrated, precipitated with cold di-ethyl ether, and filtered. The peptide mass was investigated using mass spectrometry (theoretical mass = 2718 Da; experimental mass = 2717.27 Da; ESI-TOF, Mariner System 5220, Applied Biosystem, Perkin-Elmer, Sacramento, CA, USA) to confirm the peptide identity. The peptide was purified using semipreparative RP-HPLC and analyzed through RP-HPLC using a Vydac C18 column (5 μm, 300 Å, 4.6 × 205 mm, Grace) in the following conditions: eluent A (0.05% TFA in MilliQ water); eluent B (0.05% TFA in acetonitrile); gradient, from 27 to 37% B in 20 min; flow rate, 1 mL/min; detection at 214 nm. The peptide’s retention time was 9.874 min.

### 2.4. 3D Printing of PEEK Scaffold

**Figure 1:**
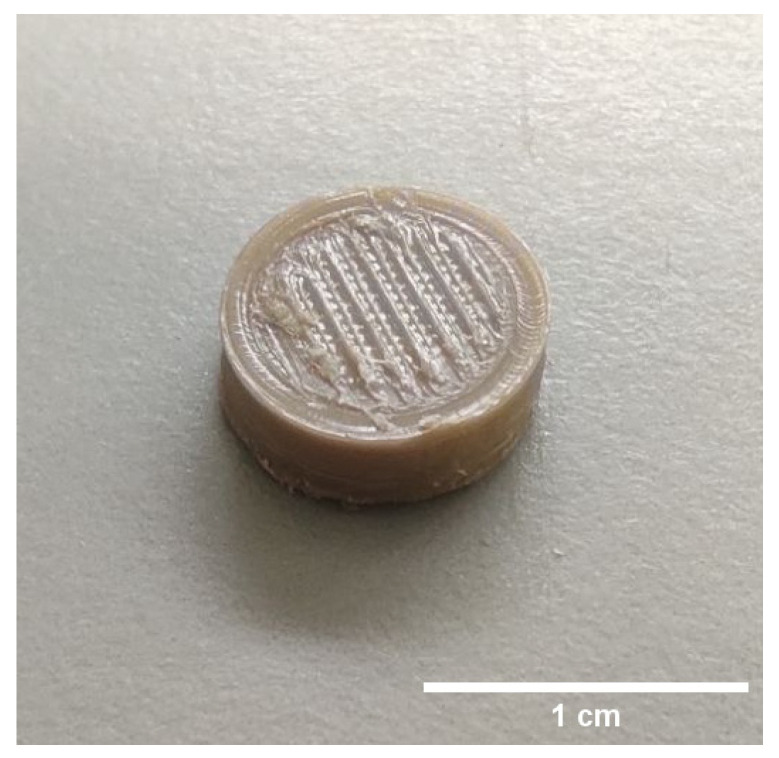
Patterned surface of 3D-printed PEEK disk.

Autodesk fusion 360 (San Rafael, CA, USA) computer-aided design (CAD) software was used to create PEEK disks files, which were then exported in *stl* format and imported into Simplify 3D software (Cincinnati, OH, USA). The files were sliced in order to generate printer-ready G code, which was then printed using an Apium P155 PEEK filament printer (Apium Additive Technologies GmBh, Willy, Karlsruhe, Germany). The printer used a PEEK filament (Vitrex Rotherham, Rotherham, UK) with a diameter of 1.75 mm and extruded it through a 0.4 mm diameter nozzle at a temperature of 485 °C onto a print bed with a temperature of 130 °C. The print speed was set to 33.3 mm/s and the resulting 3D-printed PEEK disks had a flat surface on one side and a patterned surface on the other. The PEEK disks were 10 mm in diameter and 4 mm in height, with a 100% infill. The PEEK disks were designed with a flat (lower side) and a patterned surface (upper side, Figure 1), which was obtained by extruding the PEEK filament in horizontal and vertical lines with a 0.5 mm step on the surface of the disk.

### 2.5. PEEK Surface Functionalization

All the peptides were grafted to PEEK disks.

Aoa-x-GBMP1α was bound through chemoselective ligation, whereby the amino-oxy group present on the N-terminus of the peptide chain was linked to carbonyl groups on the PEEK surface. A solution of peptide in 40 mM monobasic sodium phosphate at pH 6 was left to react with PEEK samples for 24 h at room temperature. At the end of the reaction, the samples were washed 3 times with the buffer and 3 times with MilliQ water [23]. The peptide concentration used was 10^−4^ M, since [23] proved that this concentration maximizes the cell proliferation. 

Functionalization with N_3_-x-GBMP1α occurred by dissolving the peptide in MilliQ water and leaving the solution reacting with the PEEK disks. The reaction lasted 3 h at room temperature. Then, the samples were dried out under vacuum in presence of P_2_O_5_ for 3 h. A UV lamp irradiated the dry samples for 30 min at 254 nm (distance from the lamp, 1 cm model UVSL-15 mineralight lamp 220 V, 50 Hz, 0.12 AMPS, Ultraviolet Products INC, San Gabriel, CA, USA) [26]. The functionalized disks were finally washed 2 times with acetonitrile and 2 times with MilliQ water and dried under vacuum for 1 h. The peptide concentration used was 1 mg/mL.

Samples functionalized with either Aoa-x-GBMP1α or N_3_-x-GBMP1α were called PEEK-AoaBMP or PEEK-N3BMP, respectively.

### 2.6. X-ray Photoelectron Spectroscopy (XPS) Measurements

A custom-built instrument comprising preparation and analysis ultra-high-vacuum (UHV) chambers and previously described in [29] was used to conduct XPS analysis. Prior to analysis, samples were allowed to outgas overnight in the preparation chamber at the base pressure of approximately 10^−8^ Torr before being transferred to the analysis chamber, which maintained a vacuum pressure of 10^−8^–10^−10^ Torr during measurements. The XPS investigations were conducted on flat PEEK surfaces; non-monochromatized Mg Kα (1253.6 eV) X-ray radiation was used. All the spectra were calibrated in energy, using the C 1s signal of aromatic–aliphatic carbons located at 284.7 eV binding energy (BE) as reference. After the subtraction of a Shirley-type background, curve fitting analysis of the C1s, N1s, and O1s spectra was carried out using Gaussian curves as fitting functions. The same full width at half maximum (FWHM) value was set for all individual photoemission peaks in a complex spectrum. Atomic ratio values and surface atomic concentrations were determined from peak areas, using Scofield’s atomic sensitivity factors [30].

### 2.7. Force Spectroscopy (FS) Analyses

FS analysis was conducted on non-functionalized and functionalized PEEK at room temperature to investigate the impact of peptides on the mechanical properties of interfaces. The Young’s modulus was determined from the force–distance curves collected using an NTEGRA Atomic Force Microscope (NTE-AFM, NT-MDT, Moscow, Russia) and stiff single-crystal silicon cantilevers with a symmetric tip shape (model Tap300Al-G, BudgetSensors, Bulgaria: nominal frequency 300 kHz, nominal spring constant 40 Nm^−1^, tip radius < 10 nm). The AFM measurements were carried out in triplicate, and the cantilever spring constant was measured using the Sader method [31]. To calibrate each probe, a force curve was executed on a hard-cleaned substrate (<100> silicon wafer) where no indentation took place. This calibration was necessary to determine the probe’s sensitivity and spring constant. The Derjaguin–Müller–Toporov (DMT) model [32] was utilized to compute the Young’s modulus from the experimental force–distance curves:

(1) $$F+F_{ad}=\frac{4E_{S}}{3\left(1-\nu_{s}^{2}\right)}R^{\frac{1}{2}}\delta^{\frac{3}{2}},$$

where *F* is the applied force, *F_ad_* is the adhesion force, *Es* is the Young’s modulus, *ν_s_* is the Poisson’s ratio for the sample, *R* is the radius of the spherical indenter, and *δ* is the elastic indentation depth. Each surface was indented in different areas and approximately three hundred curves were obtained, with a tip–sample approaching rate of 0.3 µms^−1^ for all force curves. The DMT model was used to fit the experimental curves in the elastic region and calculate the Young’s modulus. The distribution of the elastic modulus was analyzed using OriginPro 8.5 software. Au-coated silicon nitride tips with spring constants ranging from 0.003 to 0.13 N/m were employed to carry out adhesion force measurements. These tips were calibrated via the Sader method. The adhesion force was determined by measuring the pull-off force required to separate the AFM tip from the surface. The PEEK surfaces were probed in various regions, and approximately three hundred approach–retract cycles were recorded for each system. The tip–sample approaching velocity remained constant at 0.3 μs^−1^ for all force curves. Hooke’s law was used to calculate the adhesion force between the tip and surface, based on the cantilever deflection distance and cantilever spring constant:

F = k × ΔL (2)

where F is the force (nN), k is the spring constant of the cantilever, and ∆L is the deflection distance (nm). The cantilever spring constants were equal to 0.09 N/m for PEEK-AoaBMP and 0.07 N/m for PEEK-N3BMP.

The AFM analyses were conducted on flat PEEK surfaces.

### 2.8. Biological Assays

The 3D-printed PEEK disks were enriched with peptides on the patterned surface and utilized for all biological studies.

#### 2.8.1. Cell Culture

In order to evaluate the bioactivity of the functionalized PEEK disks, primary human osteoblasts (HOB) were utilized (Human Osteoblasts PromoCell C-12720, Heidelberg, Germany). Prior to seeding, the PEEK specimens were subjected to sterilization in 70% ethanol for 24 h and subsequently washed with PBS. Cells were cultured in Dulbecco’s modified Eagle medium (DMEM) supplemented with 10 mL of 1 M HEPES, 5 mL of 200 mM L-glutamine, 5 mL of 100× penicillin/streptomycin, 50 mL of Fetal Calf Serum (FCS), 5 mL of minimal essential medium, and 0.075 g of solid ascorbate acid. The HOB cells were microseeded at 1 × 10^5^ cells per disk onto the surfaces of the disks and incubated at 37 °C and 5% CO_2_ for the required duration of time or for all tests performed. 

To assess gene expression, a density of 2 × 10^5^ HOB cells per disk was employed. The culture medium was refreshed every three days to sustain optimal growth conditions.

#### 2.8.2. Live and Dead

In order to evaluate cell viability, a LIVE/DEAD^TM^ Viability/Cytotoxicity Kit (Thermo Fisher Scientific, Waltham, MA, USA) was used after 48 h of cell culture. Prior to exposure to the live/dead stain, two PBS washings were carried out. Images of the stained samples were captured with an Olympus IX51 (Olympus, Toxyo, Japan) fluorescent microscope, and ImageJ software (version 1.8.0_322) was used to merge the images of living cells (green fluorescence at 530 nm) and dead cells (red fluorescence at 600 nm) of the same location into one image for analysis.

#### 2.8.3. Scanning Electron Microscopy (SEM) Analysis

The morphology of HOB cells on the 3D-printed PEEK structures was observed using SEM (GeminiSEM 360, Zeiss, Jena, Germany) at a voltage of 10kV. Samples were fixed with 2.5% glutaraldehyde in 0.1 M sodium cacodylate buffer for at least 24 h at 4 °C, 48 h after seeding, to preserve the cell structure. Afterward, the samples were rinsed with 0.1 M sodium cacodylate buffer solution and treated with 1% osmium tetroxide in 0.1 M sodium cacodylate buffer for 1 h. The samples were then treated with 1% tannic acid in 0.1 M sodium cacodylate buffer for 1 h. After treatment, the samples were dehydrated in a series of aqueous ethanol solutions (20%, 30%, 40%, 50%, 60%, 70%, 90%, 96%, and 100%), twice for each concentration, with each treatment lasting for 5 min. Following dehydration, the samples were air-dried and sputter-coated with gold before SEM observation.

#### 2.8.4. Alizarin Red

To evaluate calcium deposition in HOB cells, alizarin red S staining was performed. alizarin red S is an anthraquinone dye that reacts with calcium, forming a birefringent alizarin red S/calcium complex. HOB cells were cultured for 21 days, after which the PEEK disks were washed in PBS, fixed in 10% formal saline for 15 min, and washed with deionized water thrice. The specimens were then treated with 2% alizarin red S solution for 15 min, followed by five washes in 50% ethanol.

The stained scaffolds were assessed using the protocol developed by Lee et al. [33]. To release the alizarin red S that had bound to the cells on the disks, 1 mL of 10% cetylpyridium chloride (CPC) solution was added to each well overnight in a 24-well plate. Aliquots (100 μL) of the supernatant were collected and measured using a microplate reader (Opsys MRTM 96-well microplate reader, Dynex Technologies, Chantilly, VA, USA) at 570 nm. A standard curve with known concentrations of alizarin red S in CPC solution was utilized for quantification. The results of cell mineralization were expressed as μmoles of calcium per well, as 1 mole of alizarin red S binds to 2 moles of calcium in an alizarin red S–calcium complex [34].

#### 2.8.5. Proliferation Assay

The AlamarBlue^TM^ assay was used to assess the proliferation of osteoblasts over a period of 21 days on functionalized and non-functionalized PEEK disks. The fluorescence values were measured at a test wavelength of 570 nm and a reference wavelength of 630 nm using a microplate reader (Opsys MRTM 96-well microplate reader, Dynex Technologies, Chantilly, VA, USA). Each sample was measured in triplicate to ensure the accuracy of the results. Data were collected at 1, 4, 7, 14, and 21 days to monitor the temporal profile of osteoblast proliferation.

#### 2.8.6. Quantitative Real-Time Polymerase Chain Reaction (qRT−PCR)

The present study aimed to investigate the expression levels of specific mRNA transcripts encoding human SRY-Box transcription factor 9 (SOX9), Runt-related transcription factor 2 (RUNX2), alkaline phosphatase (ALP), and osteocalcin (OCN) in HOB cultured on functionalized and non-functionalized PEEK after 1, 7, 14, and 21 days. Total RNA was extracted from the cells at the end of the incubation period using the SV Total RNA Isolation System kit (Promega, Milan, Italy), and any contaminating DNA was removed through DNase I digestion (Omega Bio-Tek, Norcross, GA, USA). The cDNA synthesis and subsequent polymerization were performed in a single step using the iTaq Universal SYBR Green One-Step Kit (Bio-Rad, Hercules, CA, USA), with a reaction mixture containing a 200 nM forward primer, 200 nM reverse primer, iTaq universal SyBR Green reaction mix, iScript reverse transcriptase, and 200 ng of total RNA. Real-time PCR was performed using the ABI PRISM 7700 Sequence Detection System (Applied Biosystems, Milan, Italy), and data were analyzed using the ΔΔCT method with human glyceraldehyde-3-phosphate dehydrogenase (GAPDH) serving as the reference gene. The PCR primers used in this study are listed in Table 1, and the target and reference genes were amplified with efficiencies near 100%.

#### 2.8.7. Statistical Analysis

All experiments were conducted in triplicate, and the results are presented as mean ± standard deviation. Statistical analysis and data processing were performed using Prism software (GraphPad Software 9). The alizarin red S assay results were subjected to one-way analysis of variance (ANOVA) testing, and Tukey’s multiple comparisons test was performed to compare the conditions. The AlamarBlue^TM^ assay and gene expression data were analyzed using two-way ANOVA tests followed by Tukey’s post hoc test. The significance level used in this study was 5%.

## 3. Results

### 3.1. Surface Characterization

#### 3.1.1. XPS Analysis

**Figure 2:**
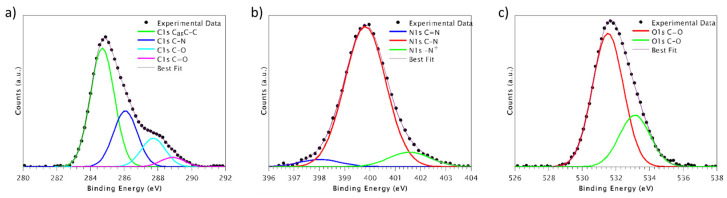
X-ray photoelectron spectroscopy results. Signals C1s, N1s, and O1s of PEEK functionalized with BMP through aminooxy chemistry are shown in (**a**–**c**), respectively.

XPS measurements were performed on all samples, specifically at the C1s, N1s, and O1s core levels. Figure 2 shows the C1s, N1s, and O1s core-level spectra of the PEEK-AoaBMP and Table 2 shows the N/C atomic ratios obtained from XPS measurements. The complete XPS data including BE, FWHM, and atomic ratios are presented in Table S1 of the Supplementary Materials; the spectra of PEEK and PEEK-N3BMP are presented in Figure S1 of the Supplementary Materials.

The C1s spectra, as shown in Figure 2a, are composed of four component peaks. The first component, which is set as a reference at 284.7 eV, is attributed to both aromatic and aliphatic carbons. The second component, observed at approximately 286.0 eV, corresponds to C-N and C-O bonds. The third one, appearing at approximately 287.6 eV, is related to the C=O bonds of PEEK, while the last component, at around 288.5 eV, is assigned to the N-C=O bonds of the immobilized peptide [35].

The N1s spectra, as illustrated in Figure 2b, consist of three components. The first component, which is located at around 398.3 eV, is attributed to the C=N nitrogen of Arg and His. The second component, appearing at approximately 399.7 eV, is associated with the C-N nitrogen of the peptide chain. The third component, at around 401.5 eV, corresponds to protonated nitrogen [36].

The O1s spectra, as presented in Figure 2c, are composed of two components. The first component, observed at approximately 531.5 eV, is attributed to C=O oxygen. The second component, at around 533.2 eV, is related to C−O oxygen.

The presence of characteristic N1s signals from the peptide indicates the immobilization of the peptide on the PEEK surface. The amount of peptide on the surface is comparable in both samples (Table 2).

#### 3.1.2. Force Spectroscopy Analysis

**Figure 3:**
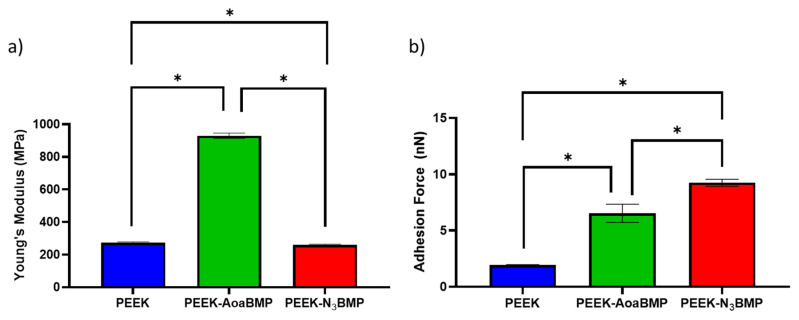
Spectroscopy analysis on non-functionalized PEEK and PEEK functionalized with GBMP1α through amino-oxy (PEEK-AoaBMP) and azido groups (PEEK-N3BMP). Surface Young’s modulus (**a**) and adhesion forces (**b**) are presented. Values are reported as mean ± SD. n = 3. * *p*-value < 0.05.

As depicted in Figure 3a, the two functionalizing techniques affect the Young’s modulus of the surface of PEEK differently. Functionalization through oxime chemistry seems to form stronger interchain interactions and a consequent almost 4-fold increase in stiffness compared to non-functionalized PEEK. On the other hand, functionalization with azido groups slightly decreased the Young’s modulus by 5%. 

In Figure 3b, the adhesion forces measured on the surfaces of PEEK samples with and without peptide functionalization are presented. The results indicate that both PEEK-AoaBMP and PEEK-N3BMP exhibited 2.27- and 4.76-fold increased tip–sample adhesion forces compared to non-functionalized PEEK, respectively. These results are due to the presence of peptides that promote intermolecular interactions with the AFM tip at the interface. 

### 3.2. Biological Tests

#### 3.2.1. Live and Dead

**Figure 4:**
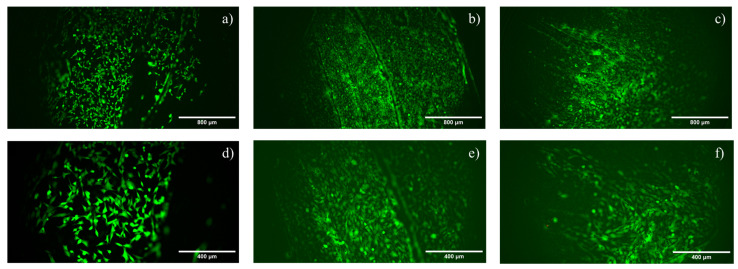
Live and dead staining of human osteoblasts cultured for 48 h on non−functionalized (**a**,**d**) and functionalized PEEK (through amino-oxy (**b**,**e**); through azido groups (**c**,**f**)). Very few dead cells (in red) are seen in (**f**) in contrast to a large number of viable cells (in green) in all the images (**a**–**f**). The magnifications used are 10× (**a**–**c**) and 20× (**d**–**f**).

Live and dead images confirm that HOB cells colonized all the test samples. Generally, on the tested surface, no dead cells (red cells) were detected after 48 h in culture, suggesting the absence of cytotoxic effects, while just a few red cells were observed on the PEEK-N3BMP sample with magnification 20× (Figure 4f, details of dead cells are reported in Figure S2 of the Supplementary Materials). The number of live cells (in green) was higher on the treated samples compared to plain PEEK (Figure 4a,d). Cells on PEEK-AoaBMP (Figure 4b,e) homogenously covered the whole surface of the scaffold.

#### 3.2.2. SEM

**Figure 5:**
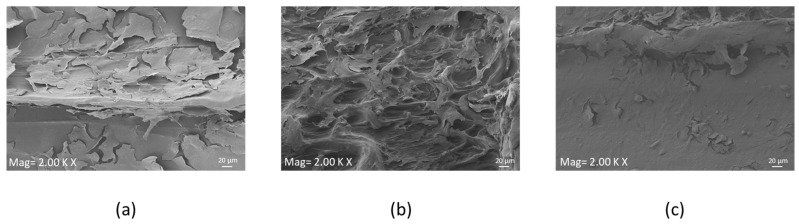
Scanning electron microscopy images of human osteoblast cells 48 h after seeding. The samples shown are the unfunctionalized PEEK sample (**a**) and PEEK functionalized with GBMP1α through amino-oxy (**b**) and azido groups (**c**). The scale bars in the images are 20 μm.

Cellular morphology and the adhesion on HOB cells were investigated on the functionalized scaffolds using SEM. Cells appeared to adhere to both the treated surfaces. On the plain PEEK sample (Figure 5a), cells appeared elongated. In contrast, Figure 5b,c shows the cells seeded on the functionalized PEEK are flattened, and they covered the whole surface, forming layers.

#### 3.2.3. Alizarin Red

**Figure 6:**
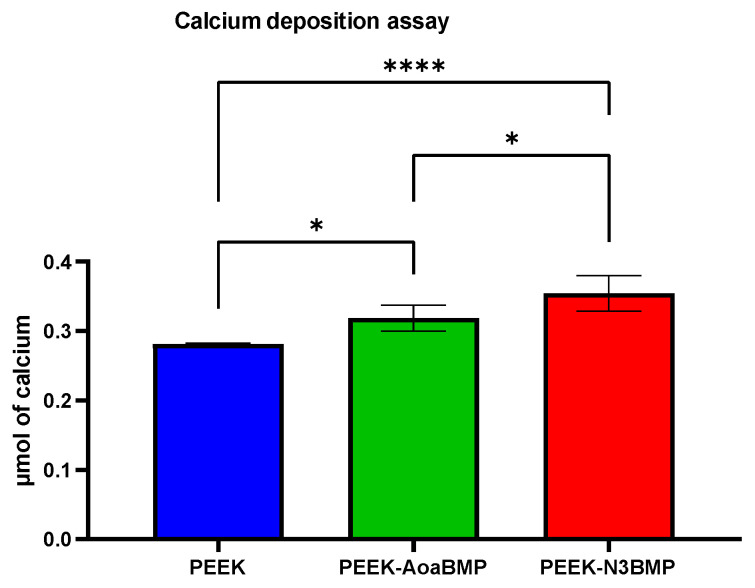
Quantification of the amount of calcium deposition through alizarin red assay 21 days after the seeding. The mineralization test was performed on non-functionalized PEEK and PEEK functionalized with GBMP1α through amino-oxy (PEEK-AoaBMP) or azido groups (PEEK-N3BMP). * *p*-value < 0.05 and **** *p*-value < 0.0001.

Both functionalization conditions statically increased mineralization after 21 days in culture (Figure 6). A greater increase in the deposition of calcium ions was observed on the PEEK−N3BMP compared to both the control and PEEK−AoaBMP. In fact, PEEK-N3BMP increased the moles of calcium on the surface by 26.1%, while PEEK-AoaBMP only increased calcium by 13.4%.

#### 3.2.4. Proliferation Assay

**Figure 7:**
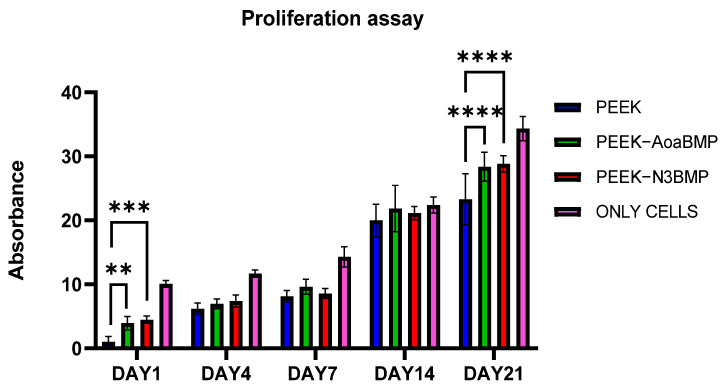
Human osteoblast proliferation evaluated on non-functionalized PEEK and PEEK functionalized with GBMP1α through amino-oxy (PEEK-AoaBMP) and azido groups (PEEK-N3BMP). AlamarBlue™ tests were assessed on days 1, 4, 7, 14, and 21 post-human osteoblast seeding. ** *p*-value < 0.01, *** *p*-value < 0.001, and **** *p*-value < 0.0001.

The proliferation of HOB cells on the functionalized PEEK samples was assessed using the AlamarBlue^TM^ assay over a period of 1, 4, 7, 14, and 21 days post-seeding. A statistically significant increase in cell proliferation was observed for both the PEEK-AoaBMP and PEEK-N3BMP samples on the first day (Figure 7). PEEK-AoaBMP had an almost 4-fold increase in proliferation on the first day 4-fold, while PEEK-N3BMP had a more than 4.5-fold increase. No significant differences were detected at the other time points, even though the proliferation was higher in functionalized samples compared to the control. On the last day assessed (day 21), both the functionalizing techniques significantly increased the proliferation by around 20%. 

#### 3.2.5. Gene Expression

**Figure 8:**
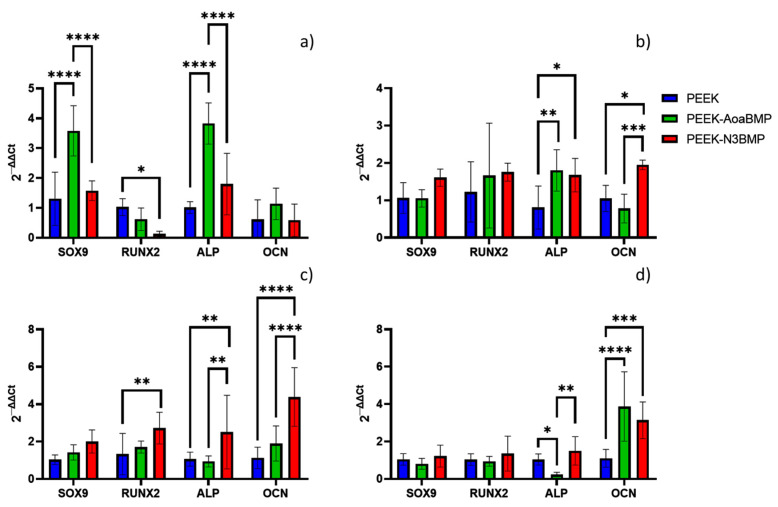
Gene expression of SRY−Box transcription factor 9 (SOX9), Runt-related transcription factor 2 (RUNX2), alkaline phosphatase (ALP), and osteocalcin (OCN) in non-functionalized PEEK and PEEK functionalized with GBMP1α through amino-oxy (PEEK-AoaBMP) and azido groups (PEEK-N3BMP). Glyceraldehyde-3-phosphate dehydrogenase (GAPDH) was used as the housekeeping gene. Relative gene expression was calculated with the $2^{-\Delta \Delta C_{t}}$ method. Time points: 1 day (**a**), 7 days (**b**), 14 days (**c**), and 21 days (**d**) from cell seeding. * *p*-value < 0.05, ** *p*-value < 0.01, *** *p*-value < 0.001, and **** *p*-value < 0.0001.

PEEK-AoaBMP induced upregulation of the SOX9 and ALP gene compared to both PEEK (3.5- and 3.8-fold increases, respectively) and PEEK-N3BMP on day 1 (Figure 8a). PEEK-AoaBMP also maintained upregulation of ALP on day 7 by 80% compared to control (Figure 8b). However, PEEK−N3BMP enhanced the expression of both genes involved in osteogenesis (ALP and OCN) by 67% and 95%, respectively. In Figure 8c, it can be seen that PEEK-N3BMP had a 2.5- and 4.3-fold increase in the rate of the upregulation of these genes, respectively. Additionally, on day 14, it overexpressed RUNX2 compared to the control by 172%. On day 21 (Figure 8d), both the functionalizing conditions gave rise to greater expression of OCN compared to unfunctionalized PEEK. PEEK-AoaBMP had a about 3.8-fold increase compared to control.

Gene expression analysis was performed to allow the detection and quantification of transcript proteins for specific genes of interest associated with bone formation. Gene regulation is an important part of cell and tissue development, with different genes being turned on and off during the different stages of development. It is well known that environmental and local factors can impact the genes expressed. Hence, in this study, the functionalized biomaterials PEEK-AoaBMP and PEEK-N3BMP were investigated for their potential to influence the enhancement of the expression of osteogenic genes.

## 4. Discussion

In this study, the covalent functionalization of PEEK’s surface with the bioactive peptide GBMP1α was realized to improve PEEK’s interactions with osteoblasts. The peptide was grafted onto the surface through two different chemical strategies: via oxime formation thanks to amino-oxy introduced in the bioactive sequence, or via the photoactivation of an azido group (present in the peptide sequence as well).

The advantages of enriching PEEK via oxime with bioactive sequences (adhesive peptides mapped from Vitronectin) have already been highlighted in our previous work [23]. The anchoring through azido is an alternative strategy assessed in order to promote a higher peptide density on the PEEK surface [26]. Although XPS detected the presence of peptide on the PEEK surface, no major difference in the outcome of functionalization with the two strategies was detected, even though the method via azido induced a slightly increased N/C ratio. The effects of PEEK modifications caused by the peptides grafted on the surface were also clear in the FS results. Increases in adhesion force were detected for both the functionalized samples with respect to bare PEEK surface, since peptides can promote intermolecular interactions with the tip of the instrument. The results are remarkably consistent with the biomechanical data relative to PEEK surface functionalized with Vitronectin-derived peptides [23]. The effects of the two different functionalizing conditions were detectable on the superficial Young’s modulus: enrichment through amino-oxy groups increased the stiffness by almost four times, while azido groups decreased it by only 5%. However, the PEEK Young’s modulus obtained through AFM and the value from the literature (about 280 MPa vs. 3.6 GPa, respectively) are different because the atomic FS used here is a method for the determination of mechanical properties on the nanoscopic scale [37], while the literature reported a mean value of the bulk polymer.

At 48 h, no cytotoxicity was induced by the peptide of the PEEK surface, as reported in live and dead assay results; a few dead cells were visualized on PEEK-N3BMP only. In fact, the functionalization of PEEK induced a better colonization by HOB cells, in particular on PEEK-AoaBMP, where cell adherence was observed to be homogeneous. Moreover, SEM showed that functionalized PEEK induced cells to adhere to the whole surface, forming layers, while the unfunctionalized PEEK did not. The visual information obtained through the live and dead and SEM assays was consistent with the proliferation tests. In fact, functionalization with GBMP1α with both amino-oxy and azido groups increased the rate of HOB proliferation. AlamarBlue^TM^ detected a significant more than 3-fold increase in the functionalized samples over the control on day 1. On days 4, 7, and 14, an increasing trend with functionalized PEEK compared to non-functionalized PEEK was observed, but this was not significant. By day 21, the proliferation rate was again significantly increased by functionalization.

To understand the role of BMP2 protein in osteogenic differentiation, we validated the expression of candidate osteogenic gene markers: SOX9, Runx2, ALP and OCN. Sox9 is an SRY-related transcription factor required for the expression of cartilaginous genes in the developing skeletal system; hence, it is associated with bone formation. RUNX2 is known to regulate the proliferation of osteoblast progenitors and their differentiation into osteoblasts. ALP, an early marker of osteogenesis, is highly expressed in the cells of mineralized tissue and plays a critical role in the formation of hard tissue. ALP acts by increasing the local rate of inorganic phosphate and facilitates mineralization, as well as reducing the extracellular pyrophosphate concentration, an inhibitor of mineral formation. OCN, a later marker of differentiation, is expressed specifically by osteoblasts, and is the most abundant non-collagenous protein in bone, influencing matrix mineralization. In this study, PEEK-AoaBMP promoted a faster and greater expression of the genes SOX9 and ALP, since on day 1, it induced significant upregulation of these genes compared to the other conditions. It has been noted that the presence of SOX9 is detectable during the process of in vitro osteogenic differentiation of human mesenchymal stem cells, enhanced by the BMP-2 protein [38], while ALP is the gene related to early-stage differentiation and calcification [39]. However, on day 7, PEEK-N3BMP had significant up-regulation in ALP (as well as PEEK-AoaBMP) and in OCN (late marker of differentiation and osteogenesis [39]). A significant increase in the expression of ALP, OCN, and RUNX2 (gene that marks early osteoblast differentiation [40]) was observed on day 14. By day 21, OCN expression was significantly up-regulated on both PEEK-AoaBMP and PEEK-N3BMP. The upregulation detected in the ALP and OCN genes involved in calcification mechanisms seems to agree with the calcium deposition results. Indeed, the alizarin red test showed a significant increase in calcium deposition on both PEEK-AoaBMP and PEEK-N3BMP compared to PEEK by 13.4% and 26.1%, respectively. Grafting through azido groups led to statistically higher results for PEEK-AoaBMP, too.

The regeneration of bone is a complex, well-orchestrated physiological process controlled by the local environment. In cases where the bone defect is large or compromised, biomaterials are often used to bridge the gap and provide a surface for the recruitment of cells. The biomaterial selection is dependent on the attitude to promote the migration, proliferation, and differentiation of bone cells, often functionalized to enhance this process. The use of PEEK has been well documented due to biocompatibility properties that avoid severe adverse reactions such as stress shielding; however, the great limitation of PEEK is its inability to integrate with the biological system. In this study, we have demonstrated that the functionalization of PEEK resulted in an enhancement of biological properties. Both chemical strategies, via oxime and via photoactivation, enhanced the activity of HOB cells on the surface of PEEK, with PEEK-N3BMP inducing a higher calcium deposition compared to PEEK-AoaBMP. 

It is well known that BMP-2 plays a significant role during embryonic development, and in bone remodeling and the maintenance of bone homeostasis in adulthood. The mechanism of action of BMP is via Smad; it acts as a signaling mediator in osteoblast–osteoclast coupling, which critically affects the rate of bone remodeling. Bone morphogenetic proteins can upregulate the expression of the inhibitory Smad proteins. These Smads are phosphorylated and translocate into the nucleus, where they regulate the transcription of target genes. A major function of BMP2 is the activation of specific osteogenic genes. However, to gain more insight and clarification on the mechanisms by which these peptides work, upcoming research will investigate their involvement in driving stem cells towards the osteogenic lineage and assess the regulatory signaling pathways in an in vitro setting.

## Figures and Tables

**Table 1:** Oligonucleotide sequences of the genes evaluated using qRT-PCR [23].

Gene	Sequence	
GAPDH	fw: 5′-acagttgccatgtagacc-3′	rv: 5′-ttgagcacagggtacttta-3′
SOX9	fw: 5′-aggaagctcgcggaccagtac-3′	rv: 5′-ggtggtccttcttgtgctgcac-3′
RUNX2	fw: 5′-cccagtatgagagtaggtgtcc-3′	rv: 5′-gggtaagactggtcataggacc-3′
ALP	fw: 5′-caacgaggtcatctccgtgatg-3′	rv: 5′-taccagttgcggttcaccgtgt-3′
OCN	fw: 5′-cgctacctgtatcaatggctgg-3′	rv: 5′-ctcctgaaagccgatgtggtca-3′

**Table 2:** X-ray photoelectron spectroscopy analysis. Quantification of N/C ratio for non-functionalized PEEK and PEEK functionalized with GBMP1α through amino-oxy (PEEK-AoaBMP) and azido groups (PEEK-N3BMP).

Sample	N/C
PEEK	/
PEEK-AoaBMP	0.14
PEEK-N3BMP	0.17

## Data Availability

Not applicable.

## Supplementary Materials

The following supporting information can be downloaded at: https://www.mdpi.com/article/10.3390/ma16103869/s1, Table S1: XPS results; Figure S1: XPS spectra and peak fit results of sample PEEK and PEEK-N3BMP; Figure S2: Live and dead staining of human osteoblasts cultured for 48 h on non-functionalized (a,d) and functionalized PEEK (through amino-oxy (b,e); through azido groups (c,f)). Only dead cells are reported here (in red). Dead cells are visible only in (f).
