# Supplementary material for: Strategies for the Covalent Anchoring of a BMP-2-Mimetic Peptide to PEEK Surface for Bone Tissue Engineering
Source: Materials (Basel). 2023 May 21;16(10):3869. doi: 10.3390/ma16103869 (PMC10222618; doi:10.3390/ma16103869)
Supplement: Supplementary file 1 [file materials-16-03869-s001.zip › materials-2372061-supplementary.pdf]

materials

MDPI

Supplementary Materials

Strategies for the Covalent Anchoring of a BMP-2-Mimetic Peptide to PEEK Surface for Bone Tissue Engineering

Table S1. XPS results.

Sample	Signal	Assignment	BE (eV)	FWHM	Internal Atomic ratios (%)	Atomic ratios
**PEEK-Controllo**	C1s	C_{ar},C-C	284.7	1.52	73	0.61
		C-O	286.3		23	0.19
		C=O	287.7		4	0.03
	O1s	C=O	531.4	1.79	51	0.08
		C-O	533.4		49	0.08
**PEEK-AoaBMP**	C1s	C_{ar},C-C	284.7	1.67	56	0.38
		C-N, C-O	286.1		26	0.18
		C=O	287.7		14	0.09
		N-C=O	288.9		4	0.03
	N1s	C=N	398.0	1.92	4	0.005
		C-N	399.8		87	0.09
		$-N^+$	401.6		9	0.009
	O1s	C=O	531.5	2.16	72	0.16
		C-O	533.1		28	0.06
**PEEK-N3BMP**	C1s	C_{ar},C-C	284.7	1.68	56	0.38
		C-N, C-O	286.1		27	0.18
		C=O	287.8		14	0.10
		N-C=O	288.7		5	0.02
	N1s	C=N	397.6	1.90	3	0.003
		C-N	399.7		90	0.10
		$-N^+$	401.7		7	0.009
	O1s	C=O	531.5	2.16	78	0.16
		C-O	533.2		22	0.04

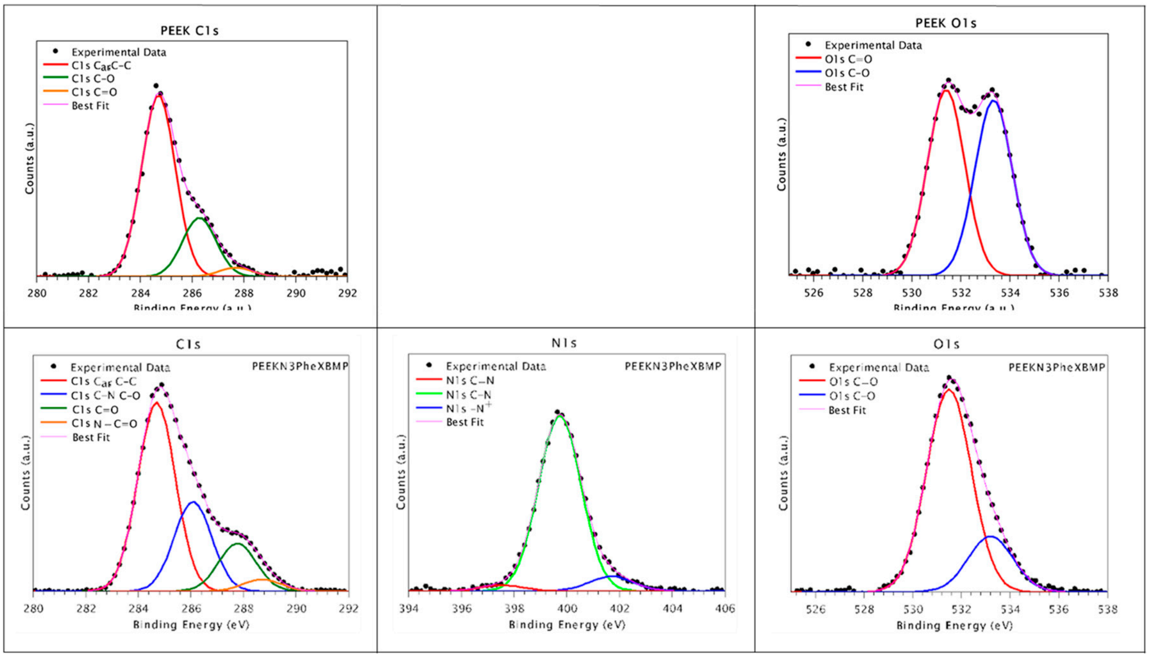

Figure S1. XPS spectra and peak fit results of sample PEEK and PEEK-N3BMP

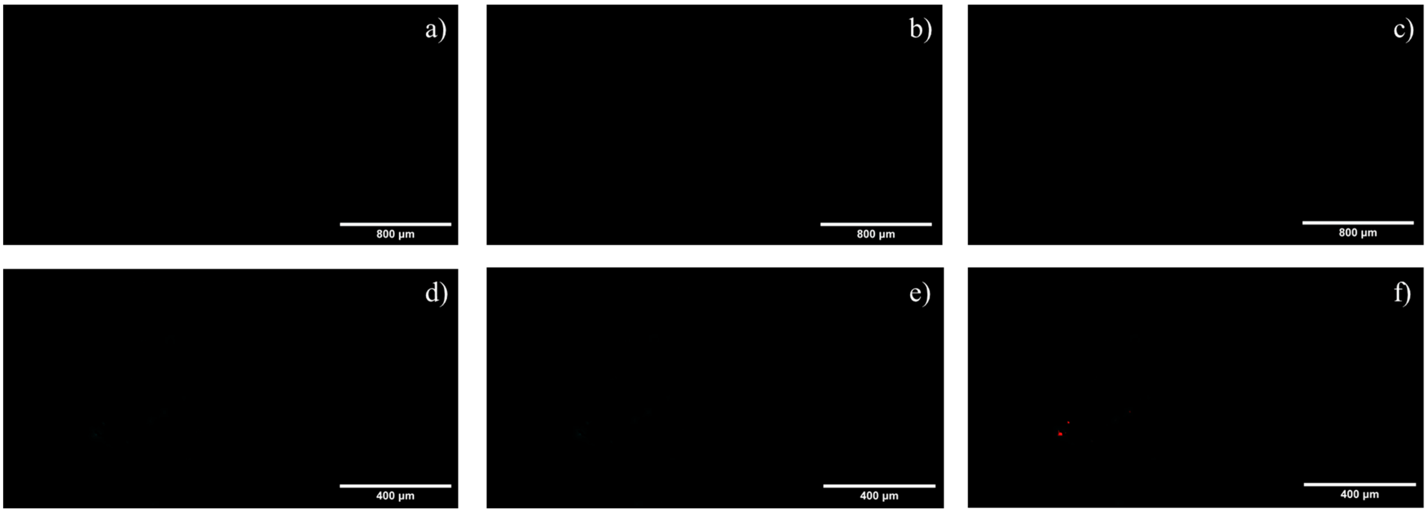

Figure S2. Live and Dead staining of human osteoblasts cultured for 48 hours on non−functionalized (a and d) and functionalized PEEK (through amino-oxy b and e; through azido group c and f). Only dead cells are here reported (in red). Dead cells are visible only in f.
